# Biochemical and Physicochemical Changes in Spaghetti Meat During Refrigerated Storage of Chicken Breast

**DOI:** 10.3389/fphys.2022.894544

**Published:** 2022-06-15

**Authors:** Giulia Tasoniero, Hong Zhuang, Brian Bowker

**Affiliations:** USDA, Agricultural Research Service, US National Poultry Research Center, Athens, GA, United States

**Keywords:** broiler chicken meat, spaghetti meat abnormality, postmortem proteolysis, calpain activity, myowater properties

## Abstract

This study investigated postmortem muscle protein degradation and myowater properties in broiler breasts afflicted with the Spaghetti Meat (SM) myopathy during 7 days of storage. Severe SM and unaffected (NORM) breast fillets were analyzed at days 0, 3, and 7 postmortem for TD-NMR myowater traits, myofibrillar protein profiles, calpain activity, free calcium, and desmin and troponin-T degradation patterns. Only at day 0, muscle histology, fiber size and sarcomere length were assessed on multiple fillet portions. In SM breasts, the intramyofibrillar water population exhibited longer relaxation times (*p* = 0.0172) and a lower proportion (*p* = 0.0118) compared to NORM. SM had a greater proportion of extramyofibrillar water (*p* = 0.0080) possessing a longer relaxation time (*p* = 0.0001). Overall, the SM myopathy had only a minor impact on the myofibrillar proteins profiles and did not affect either free calcium concentration, calpain activity, or the degradation of desmin and TnT, while storage time strongly affected all the traits measured. At microscopic level, muscle tissue from SM fillets exhibited the typical indicators of myodegeneration mostly in the superficial-cranial portion of the breast, while fiber size and sarcomere length were similar between the two muscle conditions irrespectively from the portion considered. The lack of overall significant interaction effects between muscle condition and storage period suggested that SM and NORM breast meat experience similar proteolytic and physical changes during the postmortem period.

## Introduction

Spaghetti Meat (SM) is an emerging myopathy affecting broiler chicken *Pectoralis major* muscle, whose distinguishing macroscopic trait is an overall impaired muscle integrity and a stringy, soft consistency on the ventral-cranial portion of the muscle due to poor cohesion of muscle fibre bundles (Bilgili, 2016). Although the histological features of SM are similar to those of the White Striping and Wooden Breast myopathies, SM also exhibits a progressive rarefaction of the endomysium and perimysium coupled with loose, immature connective tissue deposition around thin and split fibers (Baldi et al., 2018). These observed alterations in the connective tissue are thought to contribute to the impaired muscle integrity of SM. As the visual acceptability of the breast fillet is compromised, processors are forced to use affected meat to manufacture further-processed products (Baldi et al., 2021). Initial studies on SM focused on understanding the impact of this myopathy on basic breast meat quality and composition characteristics. The SM myopathy exerts a profound and negative impact on breast meat composition that leads to diminished functionality traits such as water-holding capacity and emulsifying properties (Baldi et al., 2018; Baldi et al., 2019; Baldi et al., 2021; Tasoniero et al., 2020). Despite having a greater pH, SM exhibits a greater proportion of extra-myofibrillar water and lower water-holding capacity compared to unaffected meat (Baldi et al., 2018; Baldi et al., 2021). Researchers observed that a diminished water-holding capacity in pork muscle is associated with decreased degradation of cytoskeletal muscle proteins both early postmortem and with increasing postmortem aging (Huff-Lonergan and Lonergan, 2005). Differently, in chicken muscle, the role of muscle protein degradation in determining muscle structural integrity and meat water-holding capacity has not been as clearly defined. In a recent study, it was suggested that a lower protein content in SM affected breasts could be related to a greater myofibrillar protein degradation accompanying the myofiber structural breakdown observed histologically (Tasoniero et al., 2020). However, little is known about the muscle protein fraction of SM or the potential impact of myofibrillar protein degradation on the muscle integrity and functional properties of SM. Thus, the purpose of this study was to investigate the effects of the SM myopathy on myofibrillar protein degradation, myowater properties, and muscle fiber characteristics. As postmortem aging is known to change some of these traits, indicators of postmortem proteolysis and myowater were measured at different postmortem times (0, 3, and 7 days postmortem). In addition, muscle histology was assessed at day 0 in order to confirm whether the histopathological lesions reflected the macroscopic assessment of the SM condition.

## Material and Methods

### Sample Collection and Preparation

During two trials, a total of 60 skinless boneless breast fillets—30 Spaghetti Meat (SM) affected and 30 unaffected (normal, NORM)—were collected at 3 h postmortem from a commercial broiler processing plant, transported to the US National Poultry Research Center (Athens, GA, United States), and subjected to 7 days of refrigerated storage. Selected SM samples exhibited a mushy and stringy consistency, due to muscle fiber bundle separation, especially in the ventral-cranial portion of the fillets (Bilgili, 2016; Baldi et al., 2018). Breasts exhibiting the white striping and/or woody breast conditions were excluded from this study. At 6–8 h post-mortem (day 0), all fillets were trimmed, weighed (NORM = 437 g, standard error = 10 vs. SM = 512 g, standard error = 10; *p* < 0.0001), and stored at 4 °C in vacuum sealed plastic bags. During each trial, analyses were carried out at 0, 3, and 7 days postmortem on five breasts (*P. Major*) per muscle condition per day. On each day of analysis, myowater properties were measured on intact fillets using time domain nuclear magnetic resonance (TD-NMR) analysis. All samples were then individually chopped in a food processor and free calcium concentration was determined in the fresh state. The remaining chopped meat was frozen in liquid nitrogen and individually stored at −80°C until myofibrillar protein, calpain, and western blot analyses were carried out. Samples for sarcomere length determination were collected only from samples processed at day 0, on knife-minced cubes of meat excised from the cranial portion of fillets (superficial and deep layers) and stored at −80°C until analysis. An additional set of five SM and five NORM fillets was collected for histological evaluation at day 0. At 24 h post-mortem, muscle samples were excised from the cranial (superficial and deep layers) and caudal portions of these fillets. Strips (approximately 0.5 cm wide, 0.5 heigh and 3–6 cm long) were cut parallel to the apparent muscle fibers, tied to a popsicle stick and frozen in isopentane chilled in liquid nitrogen, and stored at −80°C until histological analysis was performed.

### Time Domain-Nuclear Magnetic Resonance Measurements

Myowater properties of intact fillets were assessed using transverse relaxation time (*T*_*2*_) measurements collected with a LF 90II minispec NMR analyser (Bruker BioSpin, Rheinstetten, Germany). Transverse relaxation times (*T*_*2*_) were acquired using the Carr-Purcell-Meiboom-Gill (CPMG) spin-echo sequence (Carr and Purcell, 1954; Meiboom et al., 1958) (τ = 1 ms; total number of acquired echoes = 200). To obtain the relaxation time and population percentage for each of the three water populations (*T*_*2B*_, *T*_*21*_, and *T*_*22*_), the *T*_*2*_ data were analyzed using distributed exponential fitting analysis carried out with Contin software (Bruker BioSpin, Rheinstetten, Germany). Plots of relaxation amplitudes for individual relaxation processes versus relaxation times revealed the presence of the three relaxation populations (no. points of relaxation spectrum = 500; relaxation spectrum = 0.1–1,000 ms). For each of the three water populations, relaxation times were calculated from the peak position and proportions of protons exhibiting those relaxation times were calculated from the corresponding area under each peak.

### Free Calcium Concentration

To determine free calcium concentration, the procedures described by Pomponio and Ertbjerg (2012) and Soglia et al. (2018) were followed. Briefly, 25 g of chopped fresh breast meat were centrifuged at 18,000 × g for 30 min at 4°C and 5 ml of supernatant were collected. In order to provide a background ionic strength, 100 µl of 4 M KCl were added to the samples (ratio 50:1). A calibration curve was built each day of analysis, using solutions of 1,000, 100, 10, 1, and 0.1 ppm of CaCl_2_ (with background ionic strength also adjusted at a ratio 50:1) as standards. Free calcium concentration was measured in duplicate at a constant temperature (22°C ± 1 °C) with a calcium ion selective electrode equipped with a reference electrode (Orion™ Calcium 9720BNWP Electrode, Thermo Fisher Scientific, Waltham, MA, United States). Sample output in mV was converted to free calcium ppm.

### Protein Isolation and SDS-PAGE Analysis

Myofibrillar protein fractions were isolated from 2 g of minced meat according to the procedure of Pietrzak et al. (1997). A biuret assay was performed to determine protein concentrations. Samples were then diluted to a protein concentration of 2 mg/ml in sample buffer (8 mol urea, 2 mol thiourea, 3% SDS (wt/vol), 75 mmol DTT, 25 mmol Tris-HCl (pH 6.8), 0.004% bromophenol blue) and denatured for 3 min in boiling water. Denatured protein samples (15 µg protein/lane) and a broad range molecular weight standard (5–250 kDa, Thermo Scientific PageRuler Broad Range Unstained Protein Ladders, Waltham, MA, United States) were loaded onto Novex precast 4%–20% tris-glycine polyacrylamide gels (Life Technologies Corp., Carlsbad, CA, United States) and ran at 4°C at a constant voltage. Gradient gels were utilized to allow for a broader range of proteins to be analyzed, and equal protein loads ensured that differences were because of actual variations in the protein profiles. Gels were then stained (Coomassie brilliant blue R-250) and destained. The densities of 13 myofibrillar protein bands were quantified using Alpha View software (v 3.4, ProteinSimple Inc., Santa Clara, CA, United States). The relative abundance of each individual protein band was expressed as a percentage of the total protein abundance of all bands within the lane.

### Western Blot

Before performing western blots against desmin and troponin-T, electrophoretic separations were carried out as previously described with a protein load of 30 and 60 µg per lane for desmin and troponin-T, respectively. Myofibrillar proteins were transferred from SDS-PAGE gels to a 0.2 µm pore size PVDF membrane using a semi-dry blotting apparatus Trans-Blot® Turbo™ Transfer System (Bio Rad, Hercules, CA, United States). For desmin detection, membranes were soaked in blocking reagent (3% bovine serum albumin, BSA in TBS-T) for 1 h, washed in TBS-T (50 mM Tris, 150 mM NaCl, 0.5 g/L Tween-20; pH 7.5) for 5 min and soaked for 1 h on a shaker plate in a polyclonal rabbit anti-desmin primary antibody solution (Sigma-Aldrich Corp., St. Louis, MO, United States) diluted at 1:1,000 in TBS-T and 1% BSA. Membranes were then washed three times in TBS-T for 5 min each and soaked for 1 h in secondary goat anti-rabbit-IgG-AP antibody solution (Sigma-Aldrich Corp., St. Louis, MO, United States) diluted at 1:10,000 in TBS-T and washed again at the same conditions with TBS (50 mM Tris, 150 mM NaCl; pH 7.5). Membranes were then soaked in colorimetric solution (Opti-4CN diluent and substrate, Bio Rad, Hercules, CA, United States) until band development occurred. For troponin-T detection, dry membranes were re-wet with methanol, washed with phosphate buffered saline solution (PBS-T, 20 mM sodium phosphate, 150 mM NaCl, 0.1% v/v Tween-20, pH 7.4) for 5 min, and then soaked overnight in 5% blocking reagent solution (PBS-T + blocking reagent by Bio Rad, Hercules, CA, United States). Membranes were then washed five times with PBST for 10 min each and incubated for 2 h in a monoclonal mouse anti-troponin-T primary antibody solution (Sigma-Aldrich Corp., St. Louis, MO, United States) diluted at 1:1,000 in TBS-T and 1% BSA. Membranes were washed three times in PBS-T for 5 min each and then incubated with goat-anti-mouse-HRP secondary antibody diluted at 1:15,000 with PBS-T and 1% BSA. Membranes were washed three times in PBS-T for 10 min each. To amplify the reaction signal, membranes were incubated in amplification reagent (BAR, Bio-Rad Laboratories, Hercules, CA, United States) for 10 min, washed four times in 20% dimethylsulfoxide in PBS-T for 5 min each, washed two times with PBS-T for 5 min each, incubated in streptavidin-HRP diluted 1:3,000 in PBS-T and 1% BSA, and finally washed two times in PBS-T for 5 min each. For color development, membranes were incubated in Opti-4CN substrate (Bio-Rad Laboratories, Hercules, CA, United States) for 30 min under agitation. The density of immunoreactive bands was expressed as the relative abundance of each individual protein band within the lane.

### Casein Zymography

Gels for casein zymography were cast with a 12% resolving gel (30% acrylamide/bis-acrylamide 29:1, 3 M Tris-HCL pH 8.8, 0.2% casein, 10% APS, 0.08% TEMED) and a 3.5% stacking gel (30% acrylamide/bis-acrylamide 29:1, 1 M Tris-HCL pH 6.8, 10% APS, 0.12% TEMED). 6 ml of extraction buffer (100 mM Tris; 5 mM EDTA; 10 mM Monothioglycerol; pH 8.0) were added to 1 g of muscle sample (in duplicate), and samples were homogenized three times at 13,500 rpm for 20 s and centrifuged at 15,000 × g for 30 min at 4°C. A biuret assay was performed and protein concentration was adjusted to 7 mg/ml using the extraction buffer. Thereafter, 75 µl of sample were mixed with 25 µl of sample buffer (300 mM Tris, 40% glycerol, 0.02% bromophenol blue, 100 mM DTT; pH 6.8) and gels were loaded with 15 µl sample/lane. Separation was carried out at increasing voltage (80–150 V) for 4 h at 4°C in a running buffer (25 mM Tris, 192 mM glycine and 1 mM EDTA, pH 8.3). Subsequently, gels were incubated in 100 ml of incubation buffer (50 mM Tris, 10 mM monothioglycerol, 4 mM CaCl2; pH 7.5) and shaken at room temperature for 1 h, changing the buffer three times. Gels were then washed overnight in stop buffer (20 mM Tris and 10 mM EDTA, pH 7.0) and stained with EZ Blue Gel Staining (Sigma-Aldrich, St. Louis, MO, United States) until the gel absorbed all the stain and white bands appeared. Native and autolyzed calpain activity was quantified by Alpha View software (v 3.4, ProteinSimple Inc., Santa Clara, CA, United States) and expressed as the relative abundance of each individual protein band within the lane.

### Sarcomere Length

To prepare samples for sarcomere length determination, 14 ml of TX-rigor buffer (75 mM KCl, 10 mM imidazole, 2 mM MgCl_2_, 2 mM EGTA, 1 mM NaN_3,_ 0.5% Triton X-100, pH 7.2) were added to 2 g of chopped muscle samples were then homogenized twice at 18,000 rpm for 5 s and centrifuged at 1,000 × g for 10 min at 4°C. The resulting pellet was then resuspended, homogenized, and centrifuged a second time. After centrifugation, the supernatant was discarded and the pellet was resuspended with 14 ml of rigor buffer (75 mM KCl, 10 mM imidazole, 2 mM MgCl2, 2 mM EGTA, 1 mM NaN3, pH 7.2), vortexed, and centrifuged. After discarding the supernatant, the pellet was resuspended in 28 ml of rigor buffer. 5 µl of the myofibril preparation was placed on a slide with 75 µl of slide fixative (rigor buffer, 3% formaldehyde vol/vol). Slides were dried at 35°C for 10 min, rinsed with DI water, and sealed with a cover slip using 30 µl of mounting media (75 mM KCl, 10 mM Tris pH 8.5, 2 mM MgCl_2_, 2 mM EGTA, 1 mM NaN_3_, 1 mg/ml p-phenylenediamine, 75% glycerol vol/vol). Sarcomere length was assessed on 25–30 myofibrils per slide with a Carl Zeiss Axio Imager two light microscope using a 100X objective and Zen 2011 image processing software (Carl Zeiss AG, Oberkochen, Germany).

### Histology

Frozen samples were cut (10 µm thickness) transversely to muscle fiber direction using a Leica CM3050S cryostat (Leica Biosystems, Nussloch, Germany), fixed in 10% buffered formalin, and used for hematoxylin-eosin (HE) staining according to the procedure described by Warren et al. (2020). Fiber size was measured in three randomly selected fields/portion/fillet on HE stained slides using Zen 2011 image-analysis software (Carl Zeiss AG, Oberkochen, Germany). The total number of fibers measured per fillet portion was 300–450 measurements for both NORM and SM samples.

### Statistical Analysis

Statistical analysis was carried out using a mixed model (PROC MIXED) in SAS (Version 9.3, SAS Institute Inc., Cary, NC, United States). TD-NMR and free calcium content were evaluated with a two-way ANOVA mixed model considering muscle condition (M: NORM and SM), time (T: 0, 3, 7), and their interaction M × T as fixed effects and trial as a random effect. SDS-PAGE, casein zymography and Western Blot data were analyzed using the same two-way ANOVA mixed model, with the inclusion of gel among the random effects. A one-way ANOVA was used to evaluate the effect of the muscle condition on fiber size within fillet portion (considering sample ID and trial as random effects). Sarcomere length data were evaluated according to muscle condition, layer (superficial and deep), and their interaction (considering sample ID and trial as random effects). The individual fillet was considered as experimental unit for NMR, free calcium, SDS-PAGE, casein zymography and Western Blot analyses. The single fillet portion was considered as the experimental unit for histology and sarcomere length analyses. Means were separated using Bonferroni adjustments; *p* < 0.05 was assigned as significance level.

## Results and Discussion

### Histological Analyses

**FIGURE 1:**
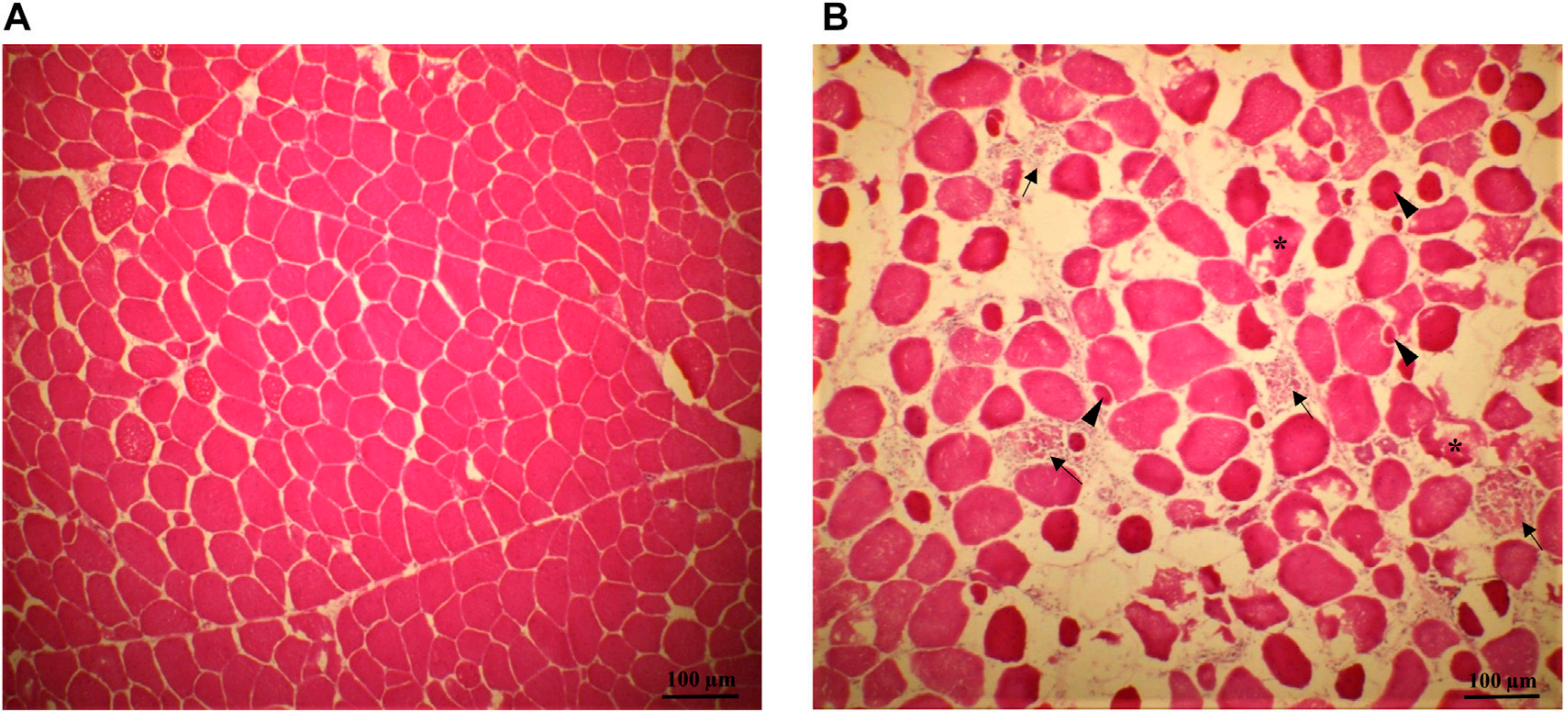
Representative pictures of transversal histological sections from the superficial portion of normal [NORM—**(A)** and Spaghetti Meat fillets (SM—**(B)**]. Muscle tissue from SM fillets exhibited many of the typical indicators of myodegeneration associated with growth related breast myopathies: muscle fibers reduced in number, spaced apart and with irregular fiber sizes; regenerative fibers (arrowheads), necrosis (asterisks), infiltration of inflammatory cells and phagocytosis of the remainder of the myofibers (arrow).

Histological analysis of the breast samples confirmed that the SM fillets utilized in this study showed the typical histopathological muscle lesions observed in previous reports (Baldi et al., 2018; Baldi et al., 2019; Mazzoni et al., 2020). Differently from the normal tissue (Figure 1A), muscle tissue from SM fillets exhibited many of the typical indicators of myodegeneration associated with growth related breast myopathies, such as irregular fiber sizes, regenerative fibers, necrosis, and infiltration of inflammatory cells (Figure 1B). In agreement with previous literature (Baldi et al., 2018), the spatial differences regarding the distribution of the lesions within the fillets reflected the macroscopic assessment of the SM condition, as the SM histopathological traits were more abundant and severe in the superficial-cranial portion of the muscle compared to the deep-cranial and caudal parts.

In this study, average muscle fiber size was not statistically different between normal and SM samples either in the superficial (NORM = 3,201 μm^2^, stdev = 1,592—vs. SM = 2,950 μm^2^, stdev = 1,480; *p* = 0.4542), deep (NORM = 3,124 μm^2^, stdev = 1,584—vs. SM = 2,861 μm^2^, stdev = 1,320; *p* = 0.5117) or caudal portions (NORM = 2,438 μm^2^, stdev = 1,337—vs. SM = 2,327 μm^2^, stdev = 1,169; *p* = 0.7002). In previous studies on myopathies, however, fibers of White Striping and Wooden Breast -affected muscles exhibited larger cross-sectional areas compared to normal tissue (Dalle Zotte et al., 2017; Mazzoni et al., 2020). Interestingly, Mazzoni et al. (2020) found that SM muscles had both larger glycolytic fibers and smaller oxidative fibers than the normal counterparts at the same time; moreover, in that study, SM possessed a lower percentage of glycolytic fibers (−8.8%) and a higher percentage of oxidative fibers than the normal counterparts (+10.2%). The lack of significance in terms of fiber size between unaffected and abnormal tissues in the current study might be explained considering that both glycolytic and oxidative fibers were included in the measurement of the average size and metabolism type was not taken into account.

### Myofibrillar Protein Profiles

**FIGURE 2:**
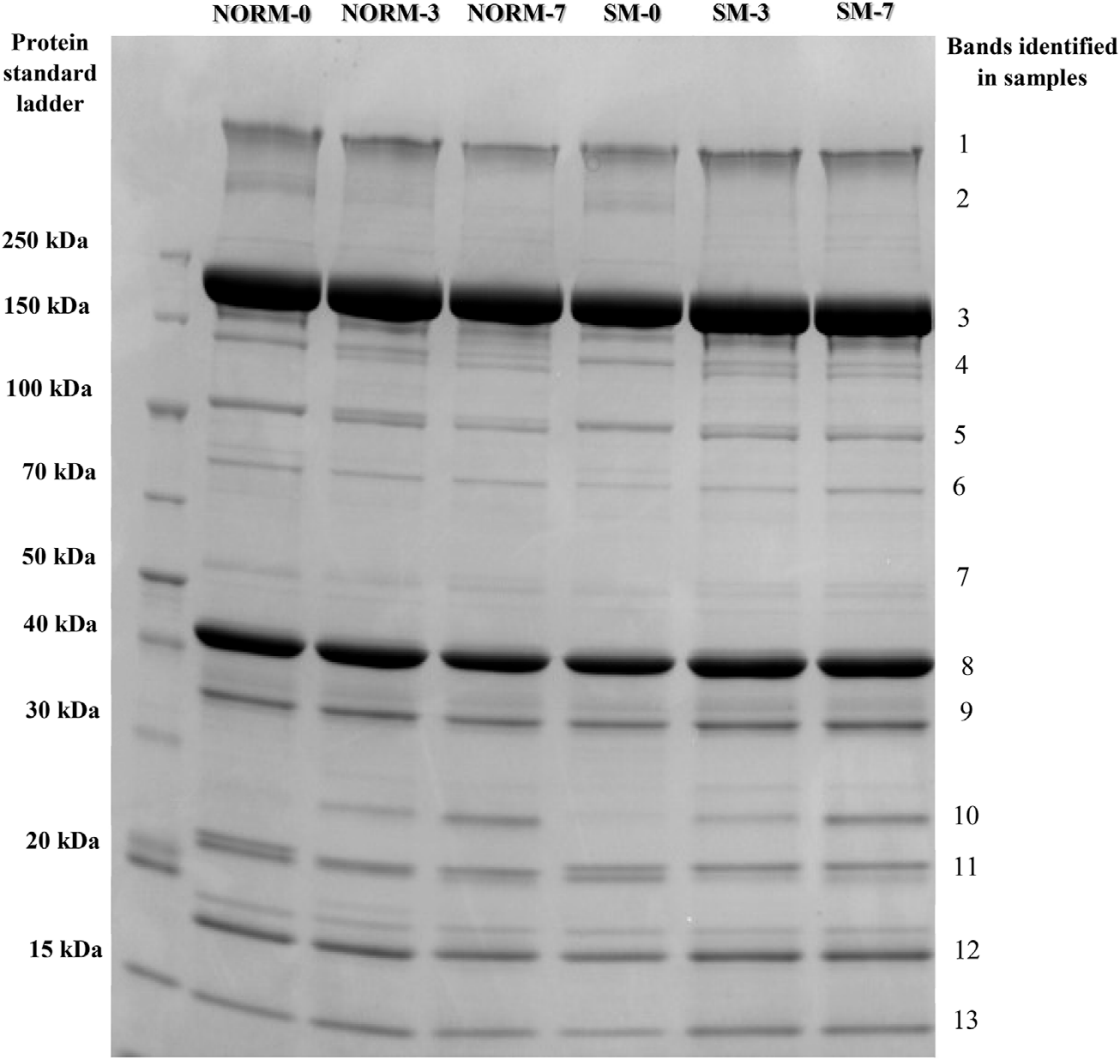
Representative SDS-PAGE protein profiles of the myofibrillar fraction according to muscle condition (M—normal, NORM; Spaghetti Meat, SM) and postmortem time (T—0, 3, 7 days).

To evaluate the effects of the SM myopathy and storage time on muscle protein composition, myofibrillar protein fractions were isolated, adjusted to equal concentrations and analysed using SDS-PAGE (Figure 2). The relative abundance of the 13 most prominent bands were quantified and shown in Table 1. The lack of significant interaction effects between muscle condition and postmortem time indicated that NORM and SM fillets exhibited a similar progression of protein degradation with postmortem storage time. Similarly, Soglia et al. (2018) reported that the myofibrillar protein degradation in Wooden Breast fillets globally occurred at the same rate as in normal fillets over a 7 day refrigerated storage period. The overall lack of significant muscle condition effects suggested that the SM myopathy had minimal impact on the myofibrillar protein profile of the breast muscle. Indeed, compared to normal fillets, SM samples only exhibited a difference in the relative abundance of a single protein band corresponding to 80 kDa (NORM = 1.00% vs. SM = 0.81%; *p* = 0.0057). Although the SM myopathy was found to reduce total muscle protein content in previous studies (Baldi et al., 2018; Baldi et al., 2019; Tasoniero et al., 2020), the electrophoretic data of the present study suggest that the myodegeneration associated with this myopathy only causes minor shifts in the myofibrillar protein profile, thereby corroborating the observations of our previous study (Tasoniero et al., 2020). Postmortem storage impacted the composition of the myofibrillar protein fraction. As the storage proceeded, the degradation of nebulin (d0 = 2.70%; d3 = 1.73%; d7 = 1.08%; *p* = 0.0001) and α-actinin (d0 = 2.42%; d3 = 2.14%; d7 = 1.74%; *p* = 0.0053) occurs concurrently with the accumulation of a 21 kDa protein band (d0 = 0.82%; d3 = 1.33%; d7 = 2.18%; *p* < 0.0001) in both normal and SM samples.

**TABLE 1:** Relative abundance^1^ of SDS-PAGE protein bands from the myofibrillar fraction of broiler breast fillets according to muscle condition (M—normal, NORM; Spaghetti Meat, SM) and postmortem time (T—0, 3, 7 days).

Bands	Approximate molecular size and identification	Muscle condition (M)		Time (T)			SE	*P*-value		
		NORM	SM	0	3	7		M × T	M	T
1	Titin	9.32	9.83	10.1	9.57	9.11	0.46	NS	NS	NS
2	Nebulin	1.85	1.83	2.70^a^	1.73^b^	1.08^b^	0.34	NS	NS	0.0001
3	220 kDa, MyHC	39.8	40.6	38.7	40.4	41.5	2.0	NS	NS	NS
4	150 kDa, MyBPC	2.46	2.30	2.95	2.24	1.94	0.48	NS	NS	NS
5	105 kDa, α-actinin	2.16	2.03	2.42^a^	2.14^ab^	1.74^b^	0.25	NS	NS	0.0053
6	80 kDa	1.00^a^	0.81^b^	0.91	0.94	0.87	0.15	NS	0.0057	NS
7	53 kDa, desmin	0.85	0.85	0.84	0.82	0.89	0.08	NS	NS	NS
8	42 kDa, actin	21.8	21.3	21.4	21.4	21.7	1.0	NS	NS	NS
9	38 kDa, TnT	5.04	5.01	5.08	5.04	4.95	0.49	NS	NS	NS
10	28–30 kDa	0.18	0.16	0.12^b^	0.27^a^	0.12^b^	0.05	NS	NS	<0.0001
11	21 kDa, MLC-1	1.55	1.34	0.82^b^	1.33^b^	2.18^a^	0.42	NS	NS	<0.0001
12	17 kDa, MLC-2	3.68	3.42	4.14^a^	3.46^b^	3.05^b^	0.43	NS	NS	0.0005
13	15 kDa, MLC-3	1.51	1.75	1.45	1.68	1.76	0.30	NS	NS	NS

1 Data expressed as individual protein band abundance as a percentage of total protein abundance in the entire lane. (MyHC, myosin heavy chain; MyBPC, myosin binding protein C; TnT, troponin T; MLC-1, myosin light chain 1; TnI, troponin I; TnC, troponin C; MLC-2, myosin light chain two; MLC-3, myosin light chain 3).

ab Means within the same row and effect followed by different superscripts differ *p* < 0.05.

### Free Calcium and Calpain Activity

**FIGURE 3:**
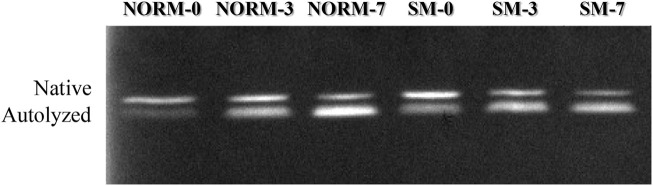
Representative casein zymogram according to muscle condition (M—normal, NORM; Spaghetti Meat, SM) and postmortem time (T—0, 3, 7 days).

**FIGURE 4:**
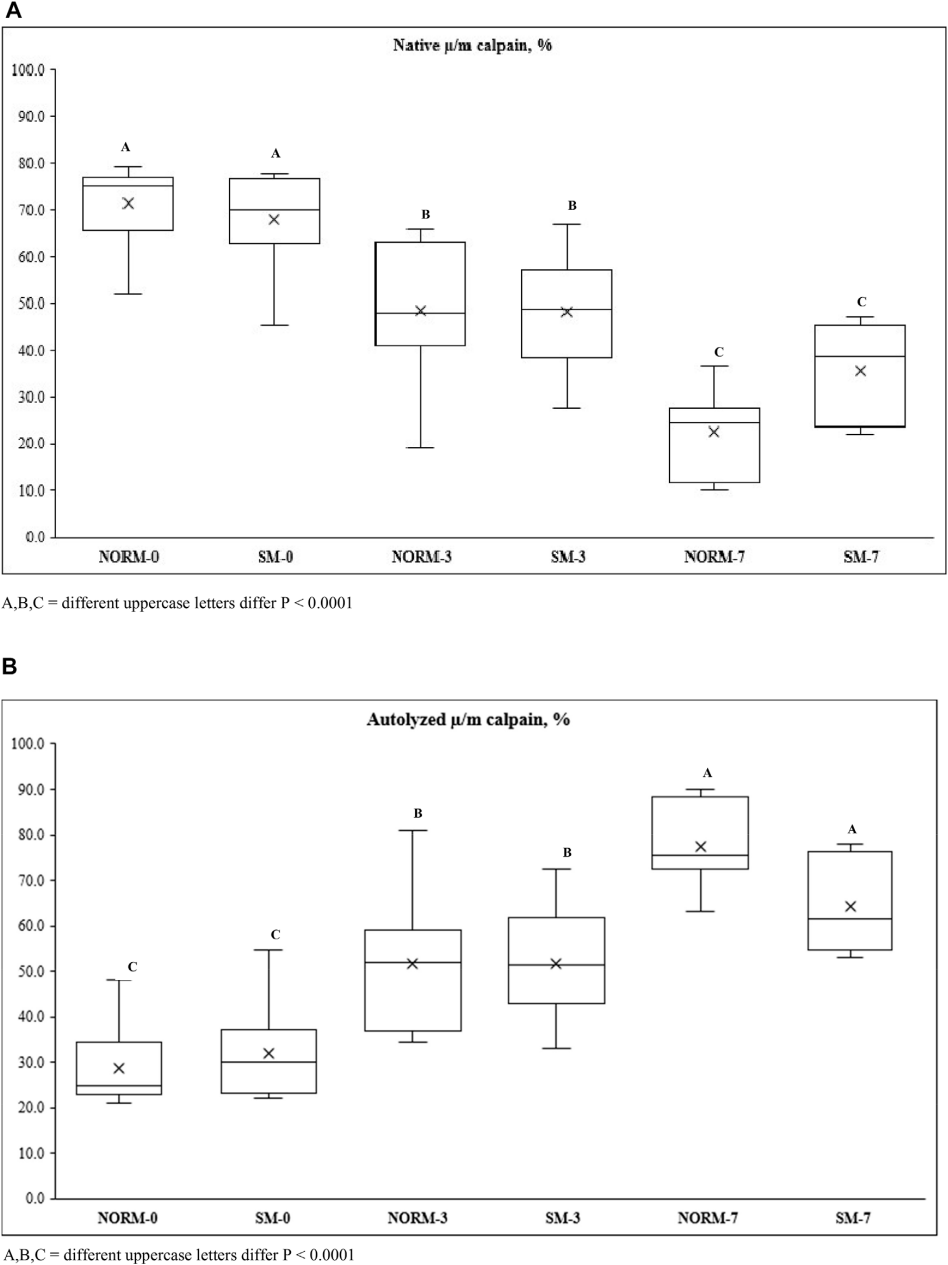
Native µ/m **(A)** and autolyzed µ/m **(B)** calpain activity of broiler breast meat according to the interaction M × T between muscle condition (M—normal, NORM; Spaghetti Meat, SM) and postmortem time (T—0, 3, 7 days).

As SDS-PAGE analysis only provides a limited view of potential myofibrillar protein degradation, free calcium content and calpain activity were measured in this study as additional indicators and precursors of proteolysis (Table 2). The interaction M × T and the SM myopathy did not significantly impact free calcium concentration in the breast meat; however, postmortem storage time exerted a strong effect on calcium levels. From day 0 to 7, the concentration of free calcium increased (d0 = 43.6 µM; d3 = 40.5 µM; d7 = 54.5 µM; *p* = 0.0392). This trend may be ascribed to a gradual leakage of calcium ions from the sarcoplasmic reticulum into the sarcoplasm (Ushio et al., 1991) as well as to the proteolysis of calcium sequestering cytoskeletal proteins (Bond and Warner, 2007). Calpain activity in the tissue was assessed using casein zymography (Figure 3). The relative proportions of both the native and autolyzed forms of µ/m calpain exhibited a significant muscle condition by storage time interaction effect (*p* = 0.0031, Table 2) and are shown in detail in Figures 4A,B. As it can be observed in the graphs, the proportions of the two forms were similar between NORM and SM fillets on days 0 and 3 postmortem, while the SM fillets tended to have a greater proportion of native µ/m calpain (and consequently a lower proportion of its autolyzed form) at day 7 postmortem. In previous literature, similar to our current observations on SM, no significant differences were observed between Wooden Breast and unaffected samples in terms of native µ/m calpain activity and the accumulation of its autolyzed form with postmortem storage time (Soglia et al., 2018). With postmortem storage time, casein zymograms demonstrated a progressive decrease in the proportion of native µ/m calpain concomitant with the accumulation of its autolyzed form. These findings are consistent with previous studies describing the activity and autolysis over time of this calpain variant in avian muscles, where it has a crucial role for the proteolysis of cytoskeletal proteins during aging (Lee et al., 2007; Huang et al., 2019).

**TABLE 2:** Free calcium content and calpain activity of broiler breast meat according to muscle condition (M—normal, NORM; Spaghetti Meat, SM) and postmortem time (T—0, 3, 7 days).

Traits	Muscle condition (M)		Time (T)			SE	*P*-value		
	NORM	SM	0	3	7		M × T	M	T
Free Ca (µM)	48.0	44.4	43.6^ab^	40.5^b^	54.5^a^	4.9	NS	NS	0.0392
Native µ/m calpain, %	48.8	49.5	70.1^a^	48.0^b^	29.4^c^	4.6	0.0031^§^	NS	<0.0001
Autolyzed µ/m calpain, %	51.2	50.5	29.9^c^	52.0^b^	70.6^a^	4.6	0.0031^§^	NS	<0.0001

ab Means within the same row and effect followed by different superscripts differ *p* < 0.05.

§ Native and autolyzed µ/m calpain activity of broiler breast meat according to the interaction M × T shown in Figure 3.

### Desmin and Troponin-T Degradation

**FIGURE 5:**
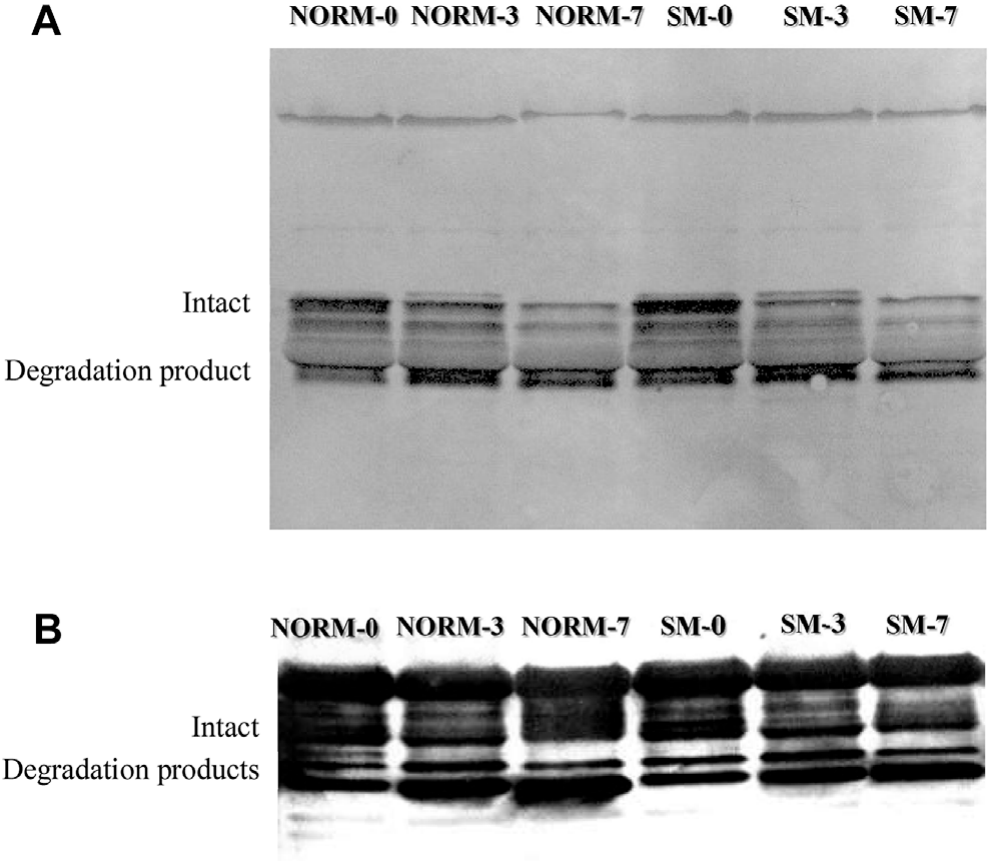
Representative western blot for desmin **(A)** and troponin-T **(B)** according to muscle condition (normal, NORM; Spaghetti Meat, SM) and postmortem time (0, 3, 7 days).

Desmin and troponin-T are often used as indicators of postmortem muscle protein degradation. In this study, western blot analysis was utilized to observe the relative abundance of desmin and troponin-T proteins (Figures 5A,B; respectively) and their degradation products in both NORM and SM fillets with postmortem storage (Table 3). Overall, the lack of significant interaction effect between muscle condition and storage time observed in the desmin and troponin-T blots (Table 3) suggested that the proteolytic processes taking place during the postmortem period are similar between SM and NORM breast meat. Due to its role in the structural organization of the sarcomere (Gallanti et al., 1992), it would have been reasonable to hypothesize that desmin accumulation may have occurred in muscle tissue during the fiber regeneration processes as a response mechanism to myodegeneration. Consistent with this hypothesis, Soglia et al. (2018) reported a larger amount of desmin in Wooden Breast samples compared to the normal ones at 10 h postmortem. Data from the current study, however, indicated that the relative abundance of intact desmin was similar between NORM and SM samples at all sampling times. This finding seems to corroborate the hypothesis that regeneration of fibres occurs in SM, but less intensely than Wooden Breast (Bilgili, 2016). Additionally, data from the current study suggest that the degree of desmin and troponin-T degradation was not affected by the occurrence of the SM condition. Consistent with the SDS-PAGE, free calcium, and calpain data, the western blot data suggest a progression of increasing myofibrillar protein degradation with postmortem aging in both NORM and SM fillets. According to Zhao et al. (2016), a strong correlation exists between µ/m calpain activity, desmin and troponin-T degradation, and the accumulation of a 28–32 kDa degradation product of troponin-T. Indeed, as a result of storage, the abundance of intact desmin decreased (d0 = 52.7%; d3 = 41.6%; d7 = 34.4%; *p* = 0.0001) and there was an accumulation of a 39-kDa degradation product (d0 = 47.3%; d3 = 58.4%; d7 = 65.7%; *p* = 0.0001). As for the degradation pattern of troponin-T, the bands ascribed to the intact form (42–40 kDa) exhibited a tendency to decrease over time (*p* = 0.0559), while protein bands ascribed to troponin-T degradation products tended to accumulate (*p* = 0.0559).

**TABLE 3:** Western Blot data for desmin and troponin-T of broiler breast meat according to muscle condition (M—normal, NORM; Spaghetti Meat, SM) and postmortem time (T—0, 3, 7 days).

Relative abundance, %	Muscle condition (M)		Time (T)			SE	*P*-value		
	NORM	SM	0	3	7		M × T	M	T
Desmin									
Intact, 52 kDa	43.8	41.9	52.7^a^	41.6^b^	34.4^b^	4.6	NS	NS	0.0001
Degradation product, 39 kDa	56.2	58.1	47.3^b^	58.4^a^	65.7^a^	4.7	NS	NS	0.0001
TnT									
Intact, 42–40 kDa	47.1	46.7	53.8	44.3	42.6	4.2	NS	NS	NS
Degradation products, 32–30 kDa	52.9	53.3	46.2	55.7	57.4	4.2	NS	NS	NS

ab Means within the same row and effect followed by different superscripts differ *p* < 0.05.

### Myowater Properties

Myowater properties of the fillets as measured by Time Domain-NMR are presented in Table 4. Within the muscle, three water populations were identified by Time Domain nuclear magnetic resonance (NMR) transverse relaxation: water tightly bound to macromolecules, water located within the myofibrillar matrix and free water held only by capillary forces. The latter population, or extramyofibrillar compartment, consists of the inter-myofibrillar spaces, as well as the spaces between muscle fibres and between fiber bundles. For each of the three compartments, the relaxation time (*T*) was measured as an indicator of water mobility. The relaxation times also provided information about population proportions (*P*), which indicated water molecule distribution. Consistent with previous literature (Baldi et al., 2018; Soglia et al., 2019; Wold and Løvland, 2020), the SM fillets possessed a lower percentage of bound water (*P*_*2B*_; NORM = 0.74% vs. SM = 0.54%; *p* = 0.0009). The intramyofibrillar water population exhibited longer relaxation times in SM fillets (*T*_*21*_; NORM = 45.9 ms vs. SM = 46.9 ms; *p* = 0.0172) and a lower proportion compared to NORM (*P*_*21*_; NORM = 83.8% vs. SM = 80.7%; *p* = 0.0118). Concurrently, SM was characterized by a greater proportion of extramyofibrillar water (*P*_*22*_; NORM = 15.5% vs. SM = 18.8%; *p* = 0.0080) possessing a longer relaxation time (*T*_*22*_; NORM = 180 ms vs. SM = 200 ms; *p* = 0.0001). According to Bertram et al. (2002), a high correlation (*r* = 0.76) exists between *P*_*22*_ population and water-holding capacity in terms of drip loss, and the correlation results increased (*r* = 0.84) when *T*_*21*_ is included in the correlation analysis. Thus, altered intramyofibrillar water properties and a greater amount of free water detected by TD-NMR in SM fillets are consistent with a lower water holding capacity previously observed in affected meat (Tasoniero et al., 2020). Altered myowater distribution and mobility in SM samples are clearly ascribable to the histological lesions observed in the affected fillets: loss of normal tissue architecture, necrosis and lysis of fibers, immature collagen deposition coupled with a progressive rarefaction of endomysial and perimysial connective tissue that leads to muscle fibers detachment from each other and compromised fiber bundles cohesion (Baldi et al., 2018). Altered myowater properties in SM might also reflect a reduced protein content exhibited by affected fillets and potentially resulting from tissue degenerative processes (Tasoniero et al., 2020). Bertram et al. (2002) found that a high correlation (*r* = 0.84) exists between *T*_*21*_ and sarcomere length. The results of the current study are in accordance with this finding, as SM also exhibited longer sarcomeres than the normal counterparts (NORM = 1.71 µm vs. SM = 1.81 µm; SE = 0.03; *p* = 0.0231). In addition, the superficial layer tended to have longer sarcomeres than the deep layer (*p* = 0.0586) while the interaction effect was not significant. Currently, the structural and functional aspects of the contractile apparatus within the SM myopathy are unknown. Similar to Wooden Breast, it might be hypothesized that longer sarcomeres are ascribable to the loss of tissue architecture that could prevent muscle shortening (Tijare et al., 2016). Postmortem storage time exerted a strong effect on myowater properties. An increasing trend for both the *P*_*2B*_ (*p* = 0.0134) and *P*_*21*_ (*p* = 0.0006) water compartments coupled with a decrease of *P*_*22*_ (*p* = 0.0005) revealed that a redistribution of myowater occurred over time in both NORM and SM fillets as a consequence of the biochemical and structural changes occurring during the storage period. Specifically, greater relative intensities of the water bound to macromolecules and located within the myofibrillar matrix might be attributed to the cytoskeletal protein proteolysis occurring during meat aging, that contribute in myofibers swelling and thus increased water holding capacity (Melody et al., 2004).

**TABLE 4:** Time Domain-Nuclear Magnetic Resonance traits of broiler breast meat according to muscle condition (M—normal, NORM; Spaghetti Meat, SM) and postmortem time (T—0, 3, 7 days).

NMR traits	Muscle condition (M)		Time (T)			SE	*P*-value		
	NORM	SM	0	3	7		M × T	M	T
*T*_*2B*_, ms	0.69	0.71	0.70	0.72	0.69	0.01	NS	NS	NS
*T*_*21*_, ms	45.9^b^	46.9^a^	47.1	45.9	46.2	0.6	NS	0.0172	NS
*T*_*22*_, ms	180^b^	200^a^	227^a^	181^b^	162^c^	6	NS	0.0001	<0.0001
*P*_*2B*_, %	0.74^a^	0.54^b^	0.59^b^	0.57^b^	0.76^a^	0.07	NS	0.0009	0.0134
*P*_*21*_, %	83.8^a^	80.7^b^	78.7^b^	83.8^a^	84.1^a^	2.1	NS	0.0118	0.0006
*P*_*22*_, %	15.5^b^	18.8^a^	20.7^a^	15.6^b^	15.0^b^	2.1	NS	0.0080	0.0005

ab Means within the same row and effect followed by different superscripts differ *p* < 0.05.

## Conclusion

The findings of the present study demonstrated that the SM myopathy only caused minor shifts in the muscle protein profile and revealed that the SM condition did not alter the initial levels of free calcium, calpain activity or the amounts of intact desmin and troponin-T. In addition, data from this study demonstrate that SM fillets do not have a greater degree of muscle protein degradation than normal fillets at 0, 3, or 7 d postmortem. These findings suggest that there is not enhanced proteolysis occurring either antemortem or early postmortem in SM that would be contributing to the soft consistency and diminished overall muscle integrity. Simultaneously, the TD-NMR data suggest that breast muscle tissue with the SM myopathy possess altered myowater properties compared to normal tissue, which is consistent with the lower WHC observed in SM fillets. Taken together, the findings in the current study on myowater properties and muscle protein degradation suggest that the differences in myowater properties observed between normal and SM fillets are not likely due to a more extensive breakdown of cytoskeletal muscle proteins in SM fillets. Further research is needed to understand more clearly how myopathy-induced changes to the physical and chemical properties of the muscle influence meat quality traits.

## Data Availability

The raw data supporting the conclusion of this article will be made available by the authors, without undue reservation.
